# Manipulation of host and parasite microbiotas: Survival strategies during chronic nematode infection

**DOI:** 10.1126/sciadv.aap7399

**Published:** 2018-03-14

**Authors:** Emily C. White, Ashley Houlden, Allison J. Bancroft, Kelly S. Hayes, Marie Goldrick, Richard K. Grencis, Ian S. Roberts

**Affiliations:** 1 School of Biological Sciences, Faculty of Biology Medicine and Health, Manchester Academic Health Science Centre, University of Manchester, Manchester M13 9PT, UK.; 2 Wellcome Trust Centre for Cell-Matrix Research, School of Biological Sciences, Faculty of Biology Medicine and Health, Manchester Academic Health Science Centre, University of Manchester, Manchester M13 9PT, UK.

## Abstract

Intestinal dwelling parasites have evolved closely with the complex intestinal microbiota of their host, but the significance of the host microbiota for metazoan pathogens and the role of their own intestinal microbiota are still not fully known. We have found that the parasitic nematode *Trichuris muris* acquired a distinct intestinal microbiota from its host, which was required for nematode fitness. Infection of germ-free mice and mice monocolonized with *Bacteroides thetaiotaomicron* demonstrated that successful *T. muris* infections require a host microbiota. Following infection, *T. muris*–induced alterations in the host intestinal microbiota inhibited subsequent rounds of infection, controlling parasite numbers within the host intestine. This dual strategy could promote the long-term survival of the parasite within the intestinal niche necessary for successful chronic nematode infection.

## Teaser

Intestinal parasite selects for its own intestinal microbiota from the host and modifies the host’s microbiota.

## INTRODUCTION

Organisms seldom live in isolation, with many forming intimate relationships with one or more species, ranging from the commensal to the parasitic. These two extremes are prevalent throughout nature, with the association between the mammalian host and the complex bacterial communities that inhabit its intestinal tract now recognized as underpinning health in its broadest sense (*1*–*3*). The microbiota of the mammalian large intestine, which contains about 10^13^ bacteria, is important for the development and modulation of the immune system and increases the metabolic potential of the host (*2*–*4*). Pathogenic organisms such as parasitic nematodes, including *Trichuris* spp., also frequently inhabit the intestinal tract. As inhabitant of the cecum and colon of a wide range of mammalian hosts, these nematodes are in close association with the surrounding host microbiota for extended periods of their host’s lives. As a consequence, coevolution of host, microbiota, and parasite has resulted in a complex interaction with important implications for each. Following the ingestion of eggs, the mouse whipworm *Trichuris muris* exploits its host’s intestinal microbiota to promote parasite egg hatching and successful establishment of infection (*5*). The subsequent infection results in marked changes to the host intestinal microbiota, which in turn affect the metabolic capabilities of the microbiota and therefore host health (*6*–*9*). We now show here that, following infection, the intestinal parasite *T. muris* first selected and then maintained its own intestinal microbiota from the infected host. We establish the importance of the *T. muris* microbiota by infecting both germ-free (GF) mice and GF mice monocolonized with *Bacteroides thetaiotaomicron* (*Bt*) and by ex vivo treatment of *T. muris* with antibiotics. All three approaches confirmed the functional importance of a *T. muris* microbiota for nematode fitness. In addition, we demonstrate functional significance to the changes that occur in the host intestinal microbiota following *T. muris* infection, namely, that the microbiota of the infected mouse suppresses subsequent parasite egg hatching, consequently controlling parasite numbers in the infected host intestine independently of the host adaptive immune system. We propose that exploitation of the host microbiota by the parasite, both as the source of its own essential microbiota and to generate an environment less permissive to egg hatching, adds a new level of complexity to the interaction between the pathogen, the host, and their respective microbiotas that is fundamental for successful chronic nematode infection.

## RESULTS

### *T. muris* selects for a distinct intestinal microbiota from the murine host

**Fig. 1:**
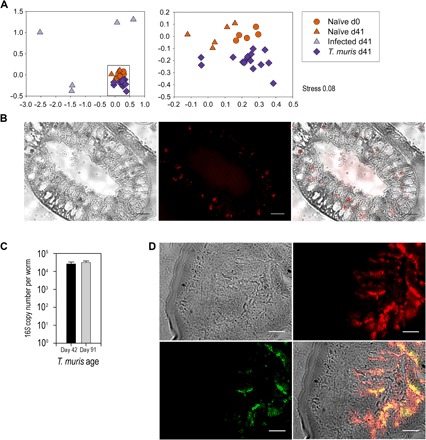
*T. muris* has an intestinal microbiota that it acquires from its host. (**A**) NMDS analysis of the rarefied operational taxonomic unit (OTU) table from 16*S* amplicon pyrosequencing comparing cecal content from control naïve mice (*n* = 5), mice with low-dose *T. muris* infection (*n* = 5), and *T. muris* internal microbiota (*n* = 15; three individual *T. muris* from each infected mouse) at day 0 (d0) and day 41 (d41). Data were collated from randomized, cohoused, and nonrandomized repeat experiments. The boxed section in the left panel is enlarged on the right. All samples are significantly different by permutational multivariate analysis of variance (PERMANOVA) tests (*P*_adjusted_ < 0.01). Axis represents scale for Euclidian distance between samples centered on zero, and stress indicates the quality of fit of data (<0.2 is a good fit). (**B**) Cross section of an adult *T. muris* intestinal tract (bright field) hybridized with a 16*S* universal Cy3 probe (red) for 2 hours and the merged image (100×). (**C**) The number of bacteria per worm was estimated by qPCR using 16*S* rRNA gene universal primers on DNA extracted from day 42 (*n* = 5) and day 91 (*n* = 5) *T. muris*. Error bars are ±SEM. (**D**) Live *T. muris* adults (bright field) were incubated overnight with GFP-expressing *E. coli* (green), washed in RPMI 1640 supplemented with penicillin/streptomycin solution, and processed for sectioning and FISH with a 16*S* universal Cy3 probe (red; 60×). Scale bars, 10 μm.

Adult-stage *T. muris* were isolated from chronically infected C57BL/6 mice at day 41 post-infection (p.i.) and were surface-sterilized so that no bacterial DNA on external surfaces was detectable by polymerase chain reaction (PCR) (fig. S1A). Next, we disrupted worm tissue, repeated PCR, and analyzed sequences of bacterial 16*S* ribosomal RNA (rRNA) genes. A parallel 16*S* rDNA analysis of cecal content from infected mice and uninfected controls at day 0 p.i. and day 41 p.i. provided data on the parasite’s bacterial environment within the host intestine before and after infection. Nonparametric multidimensional scaling (NMDS) analysis of these bacterial communities indicated the existence of a microbiota within *T. muris* worms that was significantly different from that in both the initial egg hatching environment [the naïve cecum (*P* < 0.0006)] and the environment in which the worm resided [the infected cecum (*P* < 0.001; Fig. 1A)].

Fluorescence in situ hybridization (FISH) with a Cy3-labeled universal 16*S* rRNA gene probe on sections of adult *T. muris* worms confirmed the presence of a microbiota within the nematode intestinal tract (Fig. 1B and fig. S2). Quantification of the parasite microbiota by quantitative PCR (qPCR) estimated a bacterial abundance of more than 10^4^ bacteria at day 42, similar to that observed for other nematodes such as *Caenorhabditis elegans* (*10*–*13*); bacterial abundance was maintained as the nematode aged within the host, with day 91 *T. muris* harboring similar numbers (Fig. 1C). However, embryonated *T. muris* eggs used for infections hatched in sterile conditions were free from bacteria, with no bacterial DNA detectable by PCR (fig. S1B). Therefore, *T. muris* must gain its microbiota from the naïve murine intestine it infects. To investigate the uptake and colonization of the nematode intestinal tract, we cocultured adult *T. muris* with green fluorescent protein (GFP)–expressing *Escherichia coli* and prepared them for FISH as before (Fig. 1D). The GFP *E. coli* (green) clearly localized in the intestinal tract with the resident microbiota (red) demonstrating that *T. muris* can ingest bacteria from their environment.

### The *T. muris* microbiota differs from that of its murine host

**Fig. 2:**
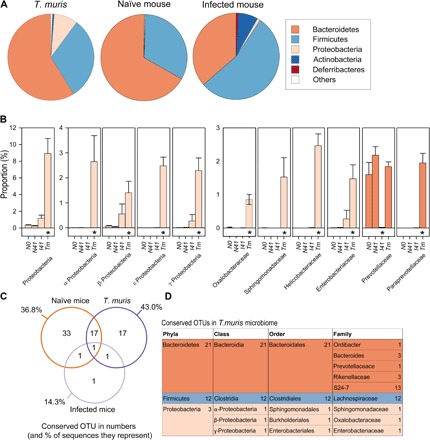
Profiling and identification of conserved OTUs in host C57BL/6 naïve and infected host intestinal microbiotas and the *T. muris* intestinal microbiota. (**A**) Comparisons of proportions at the phylum level between samples. Phyla representing less than 0.5% in all treatments were grouped together into “Other.” (**B**) Community abundance differences were compared at all taxonomic levels to identify significant shifts between groups. N0 (naïve day 0), N41 (naïve day 41), I41 (infected day 41), Tm (*T. muris* day *41*). Error bars are SEM. The asterisk denotes samples that are significantly different to all other samples from corrected post hoc Dunn test after false discovery rate (FDR) correction on Kruskal-Wallis test results (see table S2). (**C**) Venn diagram of OTUs present in all samples of a group and those shared between groups. See table S1 for list of shared OTUs. (**D**) Taxonomic identification of conserved OTUs found in all *T. muris* microbiotas. Data were collated from randomized, cohoused, and nonrandomized repeat experiments.

The mammalian intestinal tract is typically dominated by two main phyla: Bacteroidetes and Firmicutes (*2*). Naïve mice showed a similar pattern, with more than 97% of total sequences in each individual represented by these two phyla (Fig. 2A). Infection with *T. muris* caused significant restructuring of the host cecal microbiota, resulting in a major decrease in the proportion of Bacteroidetes, an increase in Firmicutes, and a reduction in total bacterial diversity (Fig. 2A and fig. S3), an observation also previously found with stool samples (*8*, *9*). Bacteroidetes decreases were characterized by specific reductions in the families Prevotellaceae [as previously identified and discussed (*8*)] and Rikenellaceae, taxa that are normally indicative of a healthy gastrointestinal tract (fig. S4) (*14*).

Although the major components of the *T. muris* microbiota were also Bacteroidetes and Firmicutes, the worm microbiota was more diverse and significantly different from both the host-infected and naïve cecal microbiotas (Figs. 1A and 2A and fig. S3). Nearly 9% of the *T. muris* microbiota was composed of Proteobacteria—a 31-fold increase in proportions compared to the naïve host and a 13-fold increase compared to infected host samples (Fig. 2B and figs. S3 and S4). Subgroups (α, β, ε, and γ) showed significant proportional increases in the *T. muris* microbiota, with rises in a range of Proteobacterial families and an increase in overall Proteobacterial diversity in comparison to both host cecal microbiotas (Fig. 2B and figs. S3 and S4). The enrichment of Proteobacteria in the *T. muris* microbiota is likely to be functionally important. Many Proteobacteria are facultative anaerobes capable of using any available sources of oxygen. Because *T. muris* resides within the intestinal epithelial cell layer, an environment with greater oxygen levels than the intestinal lumen (*15*), Proteobacteria could use any available oxygen within the nematode intestinal tract, making conditions more favorable for obligate anaerobic members of the *T. muris* microbiota. A similar phenomenon has been described in the initial colonization of neonates by Proteobacteria (*16*). Furthermore, other numerous significant increases in bacterial proportions were identified at the bacterial family and genus level in the *T. muris* microbiota compared with both host cecal microbiotas (fig. S4). These changes indicated that *T. muris* selects and maintains its own distinct microbiota regardless of the surrounding bacterial populations.

Conserved species were identified within the microbiota as those found in all *T. muris* samples (Fig. 2, C and D). There were 36 conserved species present in all *T. muris* samples (*n* = 15), making up nearly half of all sequences from the parasite (43%), the majority of which fell into the Lachnospiraceae family and the Bacteroidales subgroup S24-7 (Fig. 2D), a large family of predominantly uncultured but prevalent murine commensals (*17*). Naïve cecal samples also exhibited a conserved microbiota consisting of 52 OTUs, 18 of which were shared with all the *T. muris* samples including five Lachnospiraceae species (Fig. 2C and table S1). In contrast, only four species were conserved in all infected cecal samples, the bacterial communities that surround the parasite, resulting in a significant increase in β diversity (Fig. 2C, fig. S5, and table S1). This reflects the significant dysbiosis of the murine cecal microbiota as a consequence of infection, resulting from the divergence of microbial communities between infected individuals (*8*, *9*).

### The *T. muris* microbiota is not dependent on the hatching environment

Although the data suggest strong selection and maintenance by the parasite for its own intestinal microbiota, it is not clear whether microbiome composition is dependent on the hatching environment, that is, the initial intestinal microbiota of the host. To address this, we performed a number of experiments. First, we infected three different mouse strains with *T. muris*, C57BL/6, Rag2 knockout (KO), and C.B17 SCID (severe combined immunodeficient), all of which have distinct intestinal microbiotas (fig. S6A) (*18*). As predicted, *T. muris* infection induced significant changes to the host intestinal microbiota in all strains (fig. S6B). Critically, the microbiota of *T. muris* isolated from each host strain was remarkably similar and also distinct from its host’s intestinal microbiota (fig. S6B), suggesting that *T. muris* selected for a specific microbiota irrespective of the bacterial community it hatched and lived within.

**Fig. 3:**
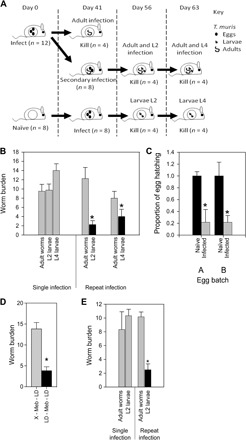
*T. muris*–induced alterations to the host intestinal microbiota significantly reduced further helminth colonization independently of the host adaptive immune system. (**A**) Experimental design for repeated *T. muris* infection. C57BL/6 mice were infected with a low dose (~20) of *T. muris* eggs at day 0 and day 41 p.i. so that mice had single or repeat infections. (**B**) Worm burdens from low-dose infections (~20 eggs) in C57BL/6 mice comparing single and repeat infections where eggs hatch in different host intestinal microbiota environments. (**C**) Cecal contents were taken from naïve and infected mice and incubated with ~130 embryonated *T. muris* eggs. Hatching rates are reported in comparison with naïve results. Egg batches, A and B, are from two separate egg harvests. (**D**) Worm burdens from low-dose infections into a naïve drug-treated host microbiota (X – Meb – LD) or a drug-treated infected microbiota void of worms (LD – Meb – LD). (**E**) Worm burdens from low-dose infections in SCID mice comparing single and repeat infections. *P < 0.002, using unpaired *t* test. Error bars are ±SEM. Mice were cohoused and randomized.

Second, C57BL/6 mice were infected with a low dose of *T. muris* (~20 eggs) on day 0, day 41, or both days to generate a repeat infection (Fig. 3A). This resulted in infected mice that had both adult stage and either L2 or L4 larval stage *T. muris* when worm burdens were subsequently assessed. Single infections with worm burdens assessed at the L2 larval stage, L4 larval stage, and adult stage were also performed. By patency (day >33 p.i.), the host intestinal microbiota is significantly changed by *T. muris* infection (*8*). As a result, *T. muris* given as a second infection at this time are exposed to an altered host intestinal microbiota, compared to those given on day 0. Community analysis identified that *T. muris* had similar microbiotas regardless of the host intestinal microbiota they infect, confirming strong selection and maintenance by the parasite (fig. S7).

### *T. muris*–induced changes in the host cecal microbiota inhibit subsequent *T. muris* infections

We observed that when *T. muris* was given as a second infection, therefore hatching in an altered host intestinal microbiota, there was a significantly reduced worm burden compared to those infecting a naïve host intestinal microbiota (Fig. 3B). This suggested that the altered host intestinal microbiota, generated as a consequence of *T. muris* infection, is an environment less favorable for egg hatching. To test this, we measured the in vitro hatching of eggs in the presence of cecal microbiota from either naïve mice or mice infected with adult-stage *T. muris* at day 35 p.i. (Fig. 3C). Hatching rates were significantly lower with cecal microbiota from infected animals, confirming that changes in the microbiota as a consequence of infection generate a microbiota less permissive for egg hatching. To further confirm this, we treated infected mice at day 35 p.i. with the anthelmintic mebendazole. This allows adult worms to be cleared while maintaining the altered infection-induced microbiota for at least 22 days after treatment, as previously shown (*8*). Hence, this provides an in vivo system to test the impact of the modified microbiota on egg hatching without the added complication of resident worms. Reinfection with eggs 10 days after mebendazole clearance resulted in a marked reduction in worm burdens compared with controls (Fig. 3D). To confirm that this effect was due to changes in the microbiota and not a consequence of the host’s immune response, we repeated this experiment in an immunodeficient C.B17 SCID mouse model, where a reduction in secondary infection worm burdens was again observed (Fig. 3E). Together, these data indicate a potential “crowd control” mechanism by the parasite where changes in the host microbiota, as a consequence of infection, generate an environment less permissible for subsequent egg hatching. This will limit further helminth colonization and ensure long-term persistence in the chronically infected individual, independent of the host adaptive immune system. To further confirm the importance of the modified microbiota on inhibiting egg hatching, GF animals were reconstituted with a cecal slurry made from chronically infected C57BL/6 mice. However, in the absence of *T. muris*, the microbiota was not stably maintained and had changed by day 14 after inoculation (fig. S8). Hence, it proved impossible to use this as a means of confirming the role of the altered microbiota in inhibiting subsequent *T. muris* infections.

### Ex vivo antibiotic treatment of *T. muris* results in a significant decline in parasite fitness

**Fig. 4:**
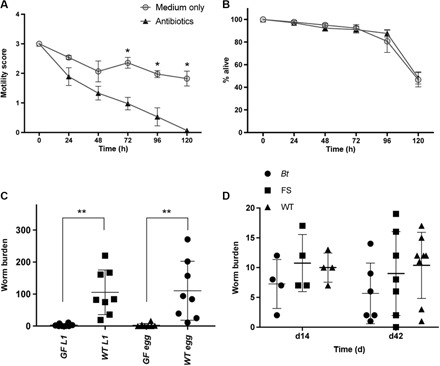
The *T. muris* microbiota is important for parasite fitness, although even a single bacterial species can enable establishment. (**A**) Adult *T. muris* worms were incubated for 5 days with an antibiotic treatment to deplete their microbiota. Medium only was used as a negative control. Motility scoring was performed every 24 hours [3, normal motility; 2, low motility (less than controls); 1, very low motility/just at one end; 0, no motility/dead]. *P < 0.0001, using analysis of variance (ANOVA). (**B**) Treatment of sterile L1 *T. muris* larvae with antibiotics or medium only as a negative control over 5 days. The number of larvae at each time point was counted, and the percentage was calculated. (**C**) Worm burdens from day 14 p.i. of GF (*n* = 8) and WT control mice (*n* = 8) infected with a high dose (~500) of sterile *T. muris* L1 larvae or a high dose of sterile eggs. **P < 0.008. (**D**) Worm burdens from low-dose infections at day 14 p.i. and day 42 p.i. of either GF animals that had been seeded with *Bt* strain VPI-5482 bacteria or GF animals that had been seeded with bacteria from a naïve C57BL/6 animal (FS, fecal slurry). C57BL/6 mice were used as infection controls (WT). Error bars are ±SEM. Experimental design precluded cohousing.

To determine whether the *T. muris* microbiota is necessary for parasite fitness, we incubated ex vivo live adult *T. muris* with a combination of antibiotics to fully deplete their microbiota in vitro. Exposure to antibiotics caused a significant decline in parasite health and consequently resulted in death, compared with *T. muris* kept in medium only (Fig. 4A). To ensure that the reduction in viability we saw was not due to an anthelmintic effect of the antibiotics, we incubated in vitro–hatched L1 larvae that lack a microbiota in the same antibiotic conditions. Antibiotics had no effect on the viability of L1 larvae compared to controls (Fig. 4B). Although we cannot rule out that developmental changes in going from L1 to adult worms somehow render the worm sensitive to antibiotics, we think that this much more likely reflects the absence and need for an internal microbiota in the L1 worm and demonstrates the importance of its own microbiota in the fitness of adult worms. To test this in vivo, GF C57BL/6 mice and wild-type (WT) controls were infected with a high dose (~500) of sterile L1 *T. muris* larvae or surface-sterilized eggs. Worm burdens were assessed at the L2 larval stage (day 14 p.i.). In contrast to WT controls, the GF mice had barely detectable worm burdens (Fig. 4C), confirming that a resident host cecal microbiota was required for both hatching of the eggs and subsequent development of L1 larvae.

### A single commensal bacterium constitutes the minimum host microbiota necessary for *T. muris* infection

To identify the minimum microbiota requirement for adult *T. muris* worms, GF mice were either monocolonized with *Bt* strain VPI-5482 or given a fecal transplant originating from uninfected C57BL/6 mice (fig. S9). *Bt* was chosen for a number of reasons: It is a major commensal component of both human and mouse intestinal microbiotas (*19*–*21*), it is routinely used in monocolonization experiments of GF mice and has been shown to re-establish multiple parameters of gut physiology and intestinal immune phenotype (*21*), and importantly, it was not identified within the *T. muris* microbiota.

*Bt*-colonized mice and those given a fecal transplant were infected with a low dose (~20) of *T. muris* eggs. Parasites developed through to patency in both cases and infection levels were comparable to C57BL/6 WT controls (Fig. 4D). In addition, a comparable parasite-specific immunoglobulin G2a/c (IgG2a/c) response was mounted in all groups (fig. S10). This indicates that, in this case, *Bt* is able to function as a surrogate worm microbiota and provide the essential factors necessary for worm development and for the host to respond immunologically as WT mice. Of course, in nature, the parasite will never encounter an intestinal environment occupied by a single bacterial species, so it will select the necessary microbiota from the ecosystem it finds itself in. Our observation that the worm microbiota was highly similar, regardless of the genotype of the infected mouse host, is consistent with the notion that a common set of requirements are important to the parasite, and that these requirements may be met by a small number of bacterial species. Although at this stage we cannot state unequivocally what constitutes a core microbiota for *T. muris*, future studies with increased numbers of worms should allow us to do so. Our discovery that *Bt* can function in this capacity coupled with the genetic tractability of *Bt* (*22*) offers an exciting route to begin to precisely define the contribution of the microbiota to the parasite and to understand the changes in the host microbiota following *T. muris* infection that inhibit subsequent *T. muris* infections.

## DISCUSSION

Research on the evolutionary relationships among host, microbiota, and macrofauna (the parasites that can inhabit our intestinal tract) has so far focused on the consequences of parasite infection for the mammalian host and its intestinal microbiota. It is clear that intestinal parasites, such as *Trichuris*, can alter their surroundings within the host intestinal tract, resulting in significant restructuring of bacterial communities and modulation of immune responses, an advantage to the parasite, controlling its environment and sustaining the health of its host. This relationship must now be expanded to include the bacterial communities that inhabit the parasite intestinal tract. We have demonstrated the importance of a complex and diverse microbiota within the intestinal tract of the pathogenic helminth parasite *T. muris*. The conservation of a distinct microbiota within the *T. muris* intestinal tract independent of the host intestinal microbiota it infects, and therefore the range of microbial ecosystems it is exposed to, suggests that the parasite selects for a specific subset of bacteria that may be linked to its survival within the host. Few other nematode intestinal microbial populations have been described, although natural isolates of *C. elegans* contain a species-rich bacterial community within their intestine, selected from but still different from those present within their environment (*23*). The observation that microbiota from congeneric *Caenorhabditis remanei* and *Caenorhabditis briggsae* isolated from different geographical sites showed conservation at the family level suggests the importance of the host in shaping the microbiota, independently of the environment. Furthermore, mixtures of experimental microbial communities, based on those naturally found in the nematodes, were shown to enhance *C. elegans* population growth (*23*). However, extensive data using the common laboratory *C. elegans* strain N2 have clearly shown that the nematode can thrive when supplied with a single bacterial species, *E. coli* strain OP50 (*24*). In a similar manner, *T. muris* naturally selects a species-rich population whose functionality can be recapitulated by *Bt*.

Given that *T. muris* eggs hatch via a bacterially driven mechanism within the host intestinal tract and that *T. muris* worms spend most of their life cycle burrowed within the host intestinal epithelial cell layer, there are various opportunities for colonization of the parasite gut after infection (*5*). Microbiota selection could occur immediately after hatching within the intestinal lumen or within the intestinal mucosal layer protecting the underlying epithelium, both of which harbor distinct microbial communities. Further work will allow us to determine at what point *T. muris* is colonized and whether this is a passive process, that is, the first bacteria that the worm comes into contact with, or whether there is some degree of selection from the parasite host for a minimum set of requirements for its own development and survival.

Filarial parasite symbionts provide their host with various metabolites or metabolic intermediates that cannot be synthesized by the parasite host but are important for various biological processes (*25*). The *T. muris* microbiota could provide a similar service to its host promoting its health and survival, a phenomenon that is observed across host-microbe interactions in nature. There are, of course, several other functions that the *T. muris* microbiota could be performing, for example, pathogen protection via colonization resistance as is the case with the microbiota of *C. elegans* (*23*) and promotion of parasite development. In light of the data showing that *Bt* can support a successful *T. muris* infection and allow for normal development, this commensal can now be used to determine the functions of the microbiota and its interaction with the parasite host.

In addition to its own microbiota, *T. muris* simultaneously manipulates the intestinal microbiota of its host, which, in turn, inhibits further infection. Although we have used mice lacking B and T cells and performed in vitro hatching experiments to minimize the input of the host immune system, this strategy does not negate a role for either innate or adaptive immunity in controlling worm numbers to challenge infections. These observed microbiota alterations could instead delay the generation of immune-mediated worm expulsion. It is well established that infection by low numbers of infectious stages of *T. muris* (as experienced naturally) allows progression to chronic infection (*26*). Similar to most intestinal dwelling nematodes, protective immunity is stimulated by high numbers of infectious stages (*27*), which only occurs naturally after multiple repeated infection events. Thus, any condition that reduces the exposure to infectious stages will delay the generation of host protective immunity and thus promote worm survival. Observations from many intestinal nematode systems suggest that, ultimately, in most individuals, partial host protective immunity is eventually generated (*28*). Given that the mouse models used in this study have functioning innate immune systems, we have not ruled out the possibility that innate immune cells are playing a role in our proposed crowd control strategy. It has been shown that the innate immune system is critical for host-microbiota interactions, maintaining intestinal homeostasis and protecting against gut injury (*29*).

The surprising result that a single species of bacteria, albeit one that is known to re-establish multiple parameters of gut physiology, could successfully reconstitute GF mice and both initiate hatching and successfully generate a chronic infection will allow us to further dissect the precise mechanisms required for both of these processes. We do not know whether *Bt*-colonized mice are refractory to further infections, but this would certainly be a focus of further study.

In conclusion, we propose that *T. muris* uses a dual approach during infection of the host intestinal tract to manipulate the host microbiota, influencing the control of future infection levels, maintaining its own beneficial microbiota, and therefore ensuring its survival and long-term persistence within the chronically infected individual.

## MATERIALS AND METHODS

### Study design

The aim of the research was to discover whether *T. muris*, an intestinal dwelling nematode, contained an intestinal microbiota and, if so, to characterize it. In addition, the research aimed to assess the functional importance of the parasite intestinal microbiota and how it was related to that of the host intestine in which the parasite lived. Finally, the research aimed to identify how the microbiota of both parasite and host contributed to the overall intestinal ecosystem during chronic helminth infection.

All animal work was designed in consultation with University of Manchester statisticians to use the minimal possible animals in experimental groups to achieve significant results. When we had variance estimates based on previous data, we performed power calculations to calculate the minimum number of animals required. If we had no variance estimates, then we used a degrees-of-freedom approach to estimate sample size. On the basis of previously published data for low-dose *Trichuris* infections, with type 1 error fixed at 5%, with a power of 80%, using a two-tailed test with an SD of 5 and an effect size of 9, the sample size should be five animals per group. The study was concluded on confirmation of sufficient data sets that had answered our initial hypotheses with sufficient robustness to be of interest to the scientific community in terms of novelty and importance. End points were selected for each experiment on the basis of previously published data sets using appropriate experimental design and statistical analysis and, where necessary, according to appropriate legislation and guidelines. No samples were removed from the analysis. The only data removed were a result of the cleaning of the raw microbiome sequencing data. All in vitro experiments were repeated a minimum of three times and representative (or collated) data shown. All in vivo results were from a minimum of two repeated experiments and collated data shown. Exact *n* numbers were stated in the figure legends. For housing, mice were divided into individually ventilated cages with enrichment of equal size on arrival by animal care technical staff with no involvement in the study design. For a single experiment, all mice used were ideally of similar age and sex and within a weight of 2 g from each other. Cohousing of mice was used where practicable to avoid issues of microbiota convergence (*30*), and this is further clarified in the figure legends. Statistical analysis varied depending on the particular experiment carried out. For example, for comparison of continuous variables from independent groups, we used Student’s *t* test (for two groups) and one-way ANOVA (for more than two groups) followed by post hoc Student’s *t* tests for all pairwise comparisons applying Bonferroni correction for multiple testing. For some approaches, blinding and random allocation of animals to experimental groups were not possible (for example, the use of GF animals and their colonization with defined microbiota). However, in these cases, samples derived from the different experimental groups were blindly analyzed.

#### Bacterial strains, media, and growth conditions

*E. coli* (PK1162) (*5*), with GFP encoded on the chromosome, was routinely grown overnight in Luria-Bertani (LB) broth [1% tryptone, 0.5% yeast extract, and 1% NaCl (w/v)] at 37°C and shaking at 200 rpm. *Bt* strain VPI-5482 was grown in TYG (tryptone, yeast, glucose) medium, as described (*21*).

#### Parasite maintenance

Stock infections of *T. muris* were maintained in susceptible mouse strains, and adult worms were harvested at day 42 p.i. Adult worms were incubated for 4 hours or overnight, and eggs were collected. The eggs were allowed to embryonate for at least 6 weeks in distilled water, and infectivity was established by worm burden in a susceptible mouse strain. Mice were infected with 150 to 300 embryonated eggs, and worm burdens were established at day 14 or day 21 p.i. *T. muris* excretory/secretory (E/S) antigen was prepared as follows. Adult worms were cultured in RPMI 1640 supplemented with 10% fetal calf serum (v/v), 2 mM l-glutamine, penicillin (100 U/ml), and streptomycin (100 μg/ml; all Invitrogen), and E/S antigen was collected after 4 hours of incubation. The E/S antigen was pelleted to remove eggs, concentrated using a Centriprep YM-10 (Amicon), and then dialyzed against phosphate-buffered saline (PBS). Protein concentration was determined using a NanoDrop (ND-1000, Labtech).

#### Animals and sampling

Male C57BL/6, AKR (both Envigo), and male or female C.B17 SCID and Rag2 KO (both homebred) mice were housed at the University of Manchester in individually ventilated cages in groups of five with diagnostic ear punches to identify individuals. For each experiment, mice were from the same batch to control for between-batch differences in the mouse intestinal microbiota. Mice were housed in the facility for 2 weeks before the experiment to stabilize communities to new conditions and kept at 22° ± 1°C and 65% humidity with a 12-hour light-dark cycle and had free access to food and water. All animal procedures were performed under the regulations of the Home Office Scientific Procedures Act (1986), Project License 70/8127, and subject to review by the University of Manchester Animal Welfare and Ethical Review Body. The experiments conform to the Animal Research: Reporting of In Vivo Experiments guidelines.

Mice were infected at 6 to 8 weeks old by oral gavage with *T. muris* E strain. A high-dose infection using ~200 eggs or a low-dose infection using ~20 eggs was given, with comparison to uninfected naïve controls. Cecal content was collected from all mice, and individual worms collected from infected individuals were washed in RPMI 1640 (Invitrogen) supplemented with penicillin (100 U/ml) and streptomycin (100 μg/ml; Sigma). All samples were stored at −80°C until DNA extraction. For mebendazole treatment, a dose of 50 mg/kg was given. Efficacy of antihelmintic treatment was confirmed by negative fecal egg counts around 7 days after treatment together with worm burdens at the end of the experiment.

GF C57BL/6 mice were kept in the Manchester Gnotobiotic Facility (MGF) at the University of Manchester. A high dose (~500) of infective *T. muris* eggs were bleached with 40% (w/v) sodium hypochlorite for 5 min followed by three washes in RPMI 1640 (Invitrogen) and used for infections. A high dose of sterile L1 larvae were prepared as detailed below (in the “In vitro hatching of *T. muris* eggs” section) and used for infections. Sterility was confirmed by growth in LB broth overnight and PCR analysis.

For L2 and L4 larvae enumeration after infection, mice were culled at day 14 or day 21 p.i. The cecum was excised and slit open longitudinally, and the epithelium was scraped with forceps. Worms could then be individually counted. For adult worm enumeration, the cecum was again excised and slit longitudinally, but adult worms were pulled individually from the epithelium.

#### Transfer of naïve and chronic microbiota or *Bt* to GF animals

To obtain naïve or chronic microbiota for transfer, naïve or chronically infected (day 50 p.i.) mice were culled, and the ceca were excised. Working quickly to limit exposure to oxygen, cecal contents were squeezed into an eppendorf and diluted 1:10 with sterile 50% glycerol/PBS (v/v). Samples were pulsed down briefly to pellet large particulate matter, and the supernatant was aliqouted and frozen at −80°C. For gavage, samples were defrosted on ice, diluted 1:5 in standard TYG medium (*21*), and then, 200 μl containing 10^8^ colony-forming units was gavaged into individual mice as described (*21*).

#### Parasite-specific antigen enzyme-linked immunosorbent assay

Analysis of parasite-specific IgG2a/c production was carried out by capture enzyme-linked immunosorbent assay. Briefly, Immulon IV plates (Dynatech) were coated with *T. muris* E/S antigen (5 μg/ml) in carbonate/bicarbonate buffer (pH 9.6) overnight at 4°C. After blocking [3% bovine serum albumin in PBS and 0.05% Tween (w/v)], eight serial 2-fold dilutions of sera (from an initial 20-fold dilution) were added to the plates. Parasite-specific antibody was detected using biotinylated rat anti-mouse IgG2a/c (BD Pharmingen).

#### In vitro hatching of *T. muris* eggs

*T. muris* eggs were hatched to produce sterile L1 larvae using 32% sodium hypochlorite in sterile water for 2 hours at 37°C with 5% CO_2_. Eggs were washed with RPMI 1640 (Invitrogen) and incubated at 37°C with 5% CO_2_ for 4 to 5 days until they hatched. For hatching in cecal contents, mice were infected with ~20 eggs and left until day 35 p.i. Control mice were left uninfected. Guts were flushed with 3 ml of sterile PBS and centrifuged (16,000*g*) for 10 s. Supernatant was removed and 200 μl was added to 800 μl of RPMI 1640 (Invitrogen) with ~130 *T. muris* eggs in 24-well culture plates (six wells per mouse) in an anaerobic chamber at 37°C. After 24 hours, ~130 *T. muris* eggs were added to each well. Cultures were incubated anaerobically for 2.75 hours at 37°C, and then, the numbers of hatched and unhatched eggs were enumerated. The average hatching rate per mouse was then used in the analysis.

#### DNA extraction

*T. muris* were surface-sterilized using 3% sodium hypochlorite for 10 min and washed with sterile water five times. The final wash was used for PCR analysis to ensure adequate removal of external bacteria. For DNA extraction of *T. muris* and cecal contents, the protocol as detailed by Griffiths *et al.* (*31*) was used.

#### Denaturing gradient gel electrophoresis

Denaturing gradient gel electrophoresis analysis of the bacterial communities present in cecal contents and adult *T. muris* worms was performed as detailed (*8*).

#### Community profiling

454 Pyrosequencing was performed on 16*S* rRNA gene amplicons from cecal contents and *T. muris* adults as detailed (*8*), yielding an average of 19,544 sequences per sample with an average coverage of 99.8% of total species using Goods estimate (*32*). Reads were ratified to the lowest sequence sample size of 7795 sequences. Sequences have been deposited at European Nucleotide Archive under accession number PRJEB12611.

#### Fluorescence in situ hybridization

*T. muris* adults were fixed in 4% paraformaldehyde (PFA) (w/v) for 24 hours, dehydrated through an ethanol gradient, cleared in cedar wood oil, and embedded in paraffin. Sections were prepared, rehydrated using an ethanol gradient, and deparaffinized in Citroclear. Sections were probed with a universal bacterial 16*S* rRNA gene Cy3 double-labeled probe EUB338 (Cy3-5′-GCTGCCTCCCGTAGGAGT-3′-Cy3; Sigma-Aldrich). Hybridization was performed using the probe (100 pmol/ml) in a probe buffer [30% formamide (v/v), 0.9 M NaCl, 20 mM tris/HCl (pH 7.4), and 0.1% SDS (w/v)] and incubated in a humid chamber at 46°C for 2 hours. Slides were washed in hybridization buffer at 48°C [0.9 M NaCl, 20 mM tris/HCl (pH 7.4), and 0.1% SDS] and treated with 0.1% Sudan Black B (w/v) (Sigma-Aldrich) for 15 min. After washing with PBS, sections were mounted with Mowiol (Sigma-Aldrich) and examined with an Olympus BX51 upright microscope using 60× or 100× objectives. Images were captured using a CoolSNAP ES camera (Photometrics) through MetaVue Software (Molecular Devices). A specific band-pass filter for Cy3 was used together with light field. Images were then processed and analyzed using ImageJ (*33*).

#### *T. muris* and bacteria coculture

Live adult *T. muris* were harvested from AKR mice infected with a high dose, added to an overnight bacterial culture of *E. coli* PK1162, and incubated overnight at 37°C with 5% CO_2_. After washing in RPMI 1640 supplemented with penicillin (100 U/ml) and streptomycin (100 μg/ml; all Invitrogen) to remove external bacteria, *T. muris* were fixed in 4% PFA (w/v) overnight and processed for FISH as above. Ingested GFP *E. coli* were detected using the fluorescein isothiocyanate band-pass filter.

#### In vitro antibiotic assay

Live *T. muris* adults were isolated from AKR mice infected with a high dose and were washed in RPMI 1640 supplemented with penicillin (100 U/ml) and streptomycin (100 μg/ml; all Invitrogen). L1 sterile larvae were prepared as detailed above (in the “In vitro hatching of *T. muris* eggs” section). *T. muris* were incubated in RPMI 1640 with or without antibiotics (nine adult worms per treatment or ~50 L1 larvae per treatment with three biological repeats for each). Antibiotics used and their final concentrations were as follows: metronidazole (1 mg/ml), ampicillin (1 mg/ml), vancomycin (0.5 mg/ml), and neomycin (1 mg/ml). Motility was scored every 24 hours until 96 hours using a motility scale from 0 to 3 (0, dead; 1, very low motility only at one end; 2, low motility that is less than in controls; and 3, normal motility) and compared to medium-only controls for adults (*34*). L1 larvae were scored as percentage alive compared to controls every 24 hours.

#### Quantitative polymerase chain reaction

Bacterial abundance was determined by qPCR using the Applied Biosystems StepOnePlus Real-Time PCR System with SYBR Green Fast PCR Master Mix (Life Technologies) in sealed 96-well plates using universal 16*S* rRNA primers: 1369F (5′- CGGTGAATACGTTCYCGG-3′) and 1492R (5′- GGWTACCTTGTTACGACTT-3′) (Sigma-Aldrich) (*35*). For qPCR standards, DNA was extracted from *E. coli* PK1162, and PCR was performed using universal primers for the whole 16*S* rRNA gene: 27F (5′-AGAGTTTGATCCTGGCTCAG-3′) and 1492R (5′-GGTTACCTTGTTACGACTT-3′) (Sigma-Aldrich) (*36*). The consequent PCR products were purified with the MinElute PCR Purification kit (Qiagen) and quantified with a Qubit (Invitrogen), and the copy number was calculated [(template concentration (ng/μl) × 10^−9^)/650 daltons] × 6.022 × 10^23^ (Avogadro’s constant)]. Serial dilutions were performed and 10^2^ to 10^7^ copies were used for the standard curve, and a primer efficiency of ~97% was consistently achieved. *T. muris* DNA was diluted to standardize qPCRs, and a control PCR was performed with primers 27F and 1492R to ensure that DNA was of sufficient quality. Each reaction contained 10 μl of SYBR Green Master Mix, forward and reverse primers at a concentration of 200 nM each, and 2 μl of template DNA in a final volume of 20 μl. The PCR reaction conditions were initial denaturation at 90°C for 20 s, followed by 40 cycles of 95°C for 15 s and 60°C for 30 s. Melt curve analysis was performed by measuring fluorescence as temperature increased from 60° to 95°C to determine the specificity of amplification after the last cycle. Each sample was run in triplicate, and five different *T. muris* adults were used for each age group. Results were expressed as the total copy number of bacterial 16*S* rRNA genes per sample by multiplying by the total DNA concentration in each sample.

#### Statistical analysis

Multivariate analysis and microbial community diversity analysis was undertaken using the vegan (*37*) and ecodist (*38*) packages in R. NMDS was used to assess communities using Bray-Curtis dissimilarities to characterize the difference between communities. The NMDS figures were plotted in arbitrary two-dimensional space with axis indicating Euclidian distance between samples centered on zero. Stress indicates the quality of fit of Bray-Curtis dissimilarities onto two-dimensional Euclidian plots (<0.2 is a good fit). All other routine statistical techniques were undertaken in R. Graphing was undertaken using SigmaPlot (Systat Software Inc.). Significant differences (*P* < 0.05) between experimental group worm burdens were determined using the Mann-Whitney *U* test, and for Microbiome data with multiple groups, multiple Kruskal-Wallis tests were used with FDR correction and post hoc Dunn tests. Significant differences (*P* < 0.05) between experimental groups for other parameters were determined using ANOVA.

## Supplementary Material

http://advances.sciencemag.org/cgi/content/full/4/3/eaap7399/DC1

## SUPPLEMENTARY MATERIALS

Supplementary material for this article is available at http://advances.sciencemag.org/cgi/content/full/4/3/eaap7399/DC1 fig. S1. PCR analysis of *T. muris* samples with 16*S* rRNA gene primers. fig. S2. FISH using a Cy3-labeled probe (NON338) complementary to EUB338 on sections of *T. muris* adults to control for nonspecific binding. fig. S3. Shannon diversity of all bacteria and the three main phyla detected in the murine microbiota before and after infection and the *T. muris* microbiota. fig. S4. Community abundance differences were compared at all taxonomic levels to identify significant shifts between groups. fig. S5. Increase in β diversity as a result of infection in the host cacal microbiota, not seen in *T. muris.* fig. S6. NMDS analysis of host intestinal microbiotas from different mouse strains infected with a high or low dose of *T. muris* compared to uninfected controls. fig. S7. NMDS analysis of DGGE comparing the microbiota of *T. muris* isolated from C57BL/6 mice infected with a low dose of *T. muris* at day 0, day 41, or both days (a single and repeat infection). fig. S8. NMDS analysis of DGGE comparing the microbiota of GF mice that have been reconstituted with a cecal slurry from chronically infected C57BL/6 mice. fig. S9. DGGE of fecal samples from GF mice inoculated with naïve mouse cecal slurry (lanes 1 to 3), pure culture of *Bt* strain VPI-5482 (lane 4), and GF mice inoculated with *Bt* (lanes 5 to 8) 12 days after inoculation and those inoculated with *Bt* at day 35 p.i. (lanes 9 to 13). fig. S10. Parasite-specific IgG2a/c antibody in serum from low dose–infected GF mice that had been reconstituted with *Bt* strain VPI-5482, with a naïve FS from a WT C57BL/6 mouse and WT C57BL/6 control mice. table S1. Species shared between groups: naïve mice, infected mice, and *T. muris* microbiotas. table S2. *P* values and FDR-adjusted *P* values for differences in bacterial proportions at different taxonomic levels between groups.
