# Supplementary material for: Manipulation of host and parasite microbiotas: Survival strategies during chronic nematode infection
Source: Sci Adv. 2018 Mar 14;4(3):eaap7399. doi: 10.1126/sciadv.aap7399 (PMC5851687; doi:10.1126/sciadv.aap7399)
Supplement: http://advances.sciencemag.org/cgi/content/full/4/3/eaap7399/DC1 [file supp_4_3_eaap7399__index.html]

Science Advances | Science Advances

## Supplementary Materials

**This PDF file includes:**

- fig. S1. PCR analysis of *T. muris* samples with 16S rRNA gene primers.
- fig. S2. FISH using a Cy3-labeled probe (NON338) complementary to EUB338 on sections of *T. muris* adults to control for nonspecific binding.
- fig. S3. Shannon diversity of all bacteria and the three main phyla detected in the murine microbiota before and after infection and the *T. muris* microbiota.
- fig. S4. Community abundance differences were compared at all taxonomic levels to identify significant shifts between groups.
- fig. S5. Increase in β diversity as a result of infection in the host cacal microbiota, not seen in *T. muris*.
- fig. S6. NMDS analysis of host intestinal microbiotas from different mouse strains infected with a high or low dose of *T. muris* compared to uninfected controls.
- fig. S7. NMDS analysis of DGGE comparing the microbiota of *T. muris* isolated from C57BL/6 mice infected with a low dose of *T. muris* at day 0, day 41, or both days (a single and repeat infection).
- fig. S8. NMDS analysis of DGGE comparing the microbiota of GF mice that have been reconstituted with a cecal slurry from chronically infected C57BL/6 mice.
- fig. S9. DGGE of fecal samples from GF mice inoculated with naïve mouse cecal slurry (lanes 1 to 3), pure culture of *Bt* strain VPI-5482 (lane 4), and GF mice inoculated with *Bt* (lanes 5 to 8) 12 days after inoculation and those inoculated with *Bt* at day 35 p.i. (lanes 9 to 13).
- fig. S10. Parasite-specific IgG2a/c antibody in serum from low dose–infected GF mice that had been reconstituted with *Bt* strain VPI-5482, with a naïve FS from a WT C57BL/6 mouse and WT C57BL/6 control mice.
- Legends for tables S1 and S2

Download PDF

**Other Supplementary Material for this manuscript includes the following:**

- table S1 (Microsoft Excel format). Species shared between groups: naïve mice, infected mice, and *T. muris* microbiotas.
- table S2 (Microsoft Excel format). *P* values and FDR-adjusted *P* values for differences in bacterial proportions at different taxonomic levels between groups.

**Files in this Data Supplement:**

- Adobe PDF - aap7399\_SM.pdf
