# Supplementary material for: Manipulation of host and parasite microbiotas: Survival strategies during chronic nematode infection
Source: Sci Adv. 2018 Mar 14;4(3):eaap7399. doi: 10.1126/sciadv.aap7399 (PMC5851687; doi:10.1126/sciadv.aap7399)
Supplement: http://advances.sciencemag.org/cgi/content/full/4/3/eaap7399/DC1 [file aap7399_SM.pdf]

Science Advances
AAAS

advances.sciencemag.org/cgi/content/full/4/3/eaap7399/DC1

Supplementary Materials for

Manipulation of host and parasite microbiotas: Survival strategies during chronic nematode infection

Emily C. White, Ashley Houlden, Allison J. Bancroft, Kelly S. Hayes, Marie Goldrick, Richard K. Grencis, Ian S. Roberts

Published 14 March 2018, *Sci. Adv.* **4**, eaap7399 (2018)
DOI: 10.1126/sciadv.aap7399

The PDF file includes:

- fig. S1. PCR analysis of *T. muris* samples with 16*S* rRNA gene primers.
- fig. S2. FISH using a Cy3-labeled probe (NON338) complementary to EUB338 on sections of *T. muris* adults to control for nonspecific binding.
- fig. S3. Shannon diversity of all bacteria and the three main phyla detected in the murine microbiota before and after infection and the *T. muris* microbiota.
- fig. S4. Community abundance differences were compared at all taxonomic levels to identify significant shifts between groups.
- fig. S5. Increase in β diversity as a result of infection in the host cacal microbiota, not seen in *T. muris.*
- fig. S6. NMDS analysis of host intestinal microbiotas from different mouse strains infected with a high or low dose of *T. muris* compared to uninfected controls.
- fig. S7. NMDS analysis of DGGE comparing the microbiota of *T. muris* isolated from C57BL/6 mice infected with a low dose of *T. muris* at day 0, day 41, or both days (a single and repeat infection).
- fig. S8. NMDS analysis of DGGE comparing the microbiota of GF mice that have been reconstituted with a cecal slurry from chronically infected C57BL/6 mice.
- fig. S9. DGGE of fecal samples from GF mice inoculated with naïve mouse cecal slurry (lanes 1 to 3), pure culture of *Bt* strain VPI-5482 (lane 4), and GF mice inoculated with *Bt* (lanes 5 to 8) 12 days after inoculation and those inoculated with *Bt* at day 35 p.i. (lanes 9 to 13).
- fig. S10. Parasite-specific IgG2a/c antibody in serum from low dose–infected GF mice that had been reconstituted with *Bt* strain VPI-5482, with a naïve FS from a WT C57BL/6 mouse and WT C57BL/6 control mice.

- Legends for tables S1 and S2

Other Supplementary Material for this manuscript includes the following:
(available at advances.sciencemag.org/cgi/content/full/4/3/eaap7399/DC1)

- table S1 (Microsoft Excel format). Species shared between groups: naïve mice, infected mice, and *T. muris* microbiotas.
- table S2 (Microsoft Excel format). *P* values and FDR-adjusted *P* values for differences in bacterial proportions at different taxonomic levels between groups.

Supplementary Materials

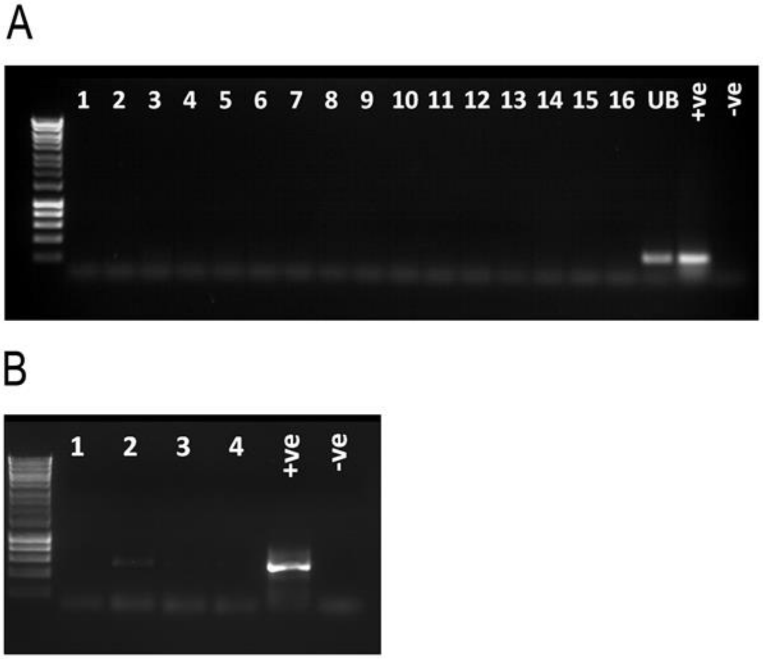

fig. S1. PCR analysis of *T. muris* samples with 16*S* rRNA gene primers. (**A**) Bleaching *T. muris* adult worms results in successful surface sterilisation. The numbers across the top denote lane number. Washes with H_2O after bleaching were used as a template for PCR (lanes 1-16) with no detectable DNA. Lane UB is a wash from an unbleached *T. muris* adult, +ve is *E. coli* genomic DNA as a positive control and –ve is H_2O as a negative control. (**B**) Embryonated eggs and L1 larvae used for infections are free from bacteria. Faint band detected in '2' was sequenced and is *T. muris* DNA.

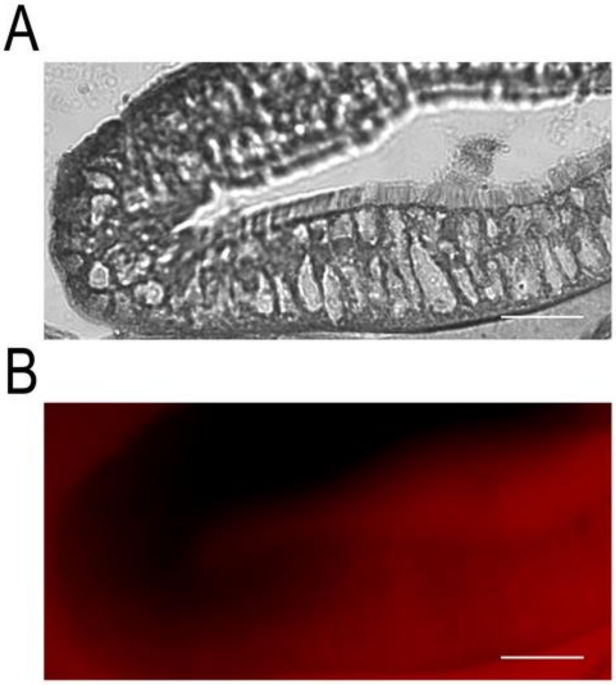

fig. S2. FISH using a Cy3-labeled probe (NON338) complementary to EUB338 on sections of *T. muris* adults to control for nonspecific binding. Sections were imaged using the (**A**) bright field or (**B**) Cy3 channels (100X). Scale bars, 10 µm.

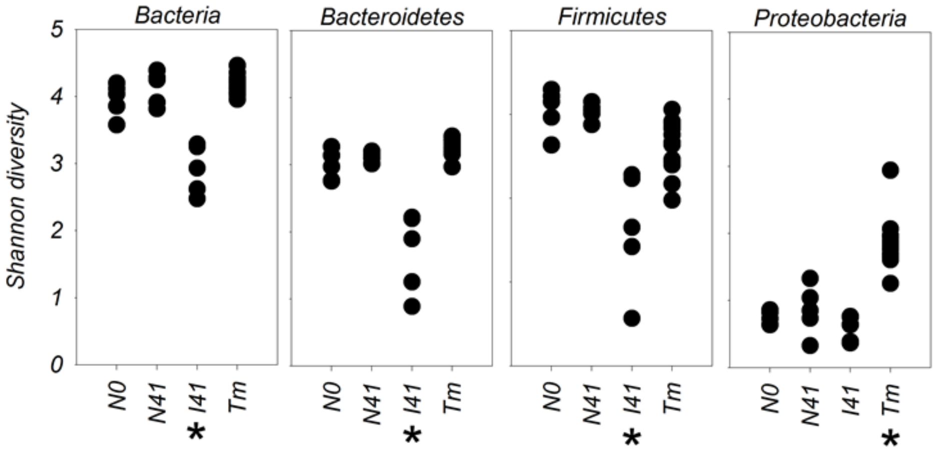

fig. S3. Shannon diversity of all bacteria and the three main phyla detected in the murine microbiota before and after infection and the *T. muris* microbiota. N0= Naïve cecal contents day 0, N41 = Naïve cecal contents day 41 p.i., I41 = Infected cecal contents day 41 p.i., Tm = *T. muris* microbiota day 41 p.i., * denotes samples are significantly different to all others tested by post hoc TukeyHSD on ANOVA results ($p < 0.001$). Samples are normally distributed.

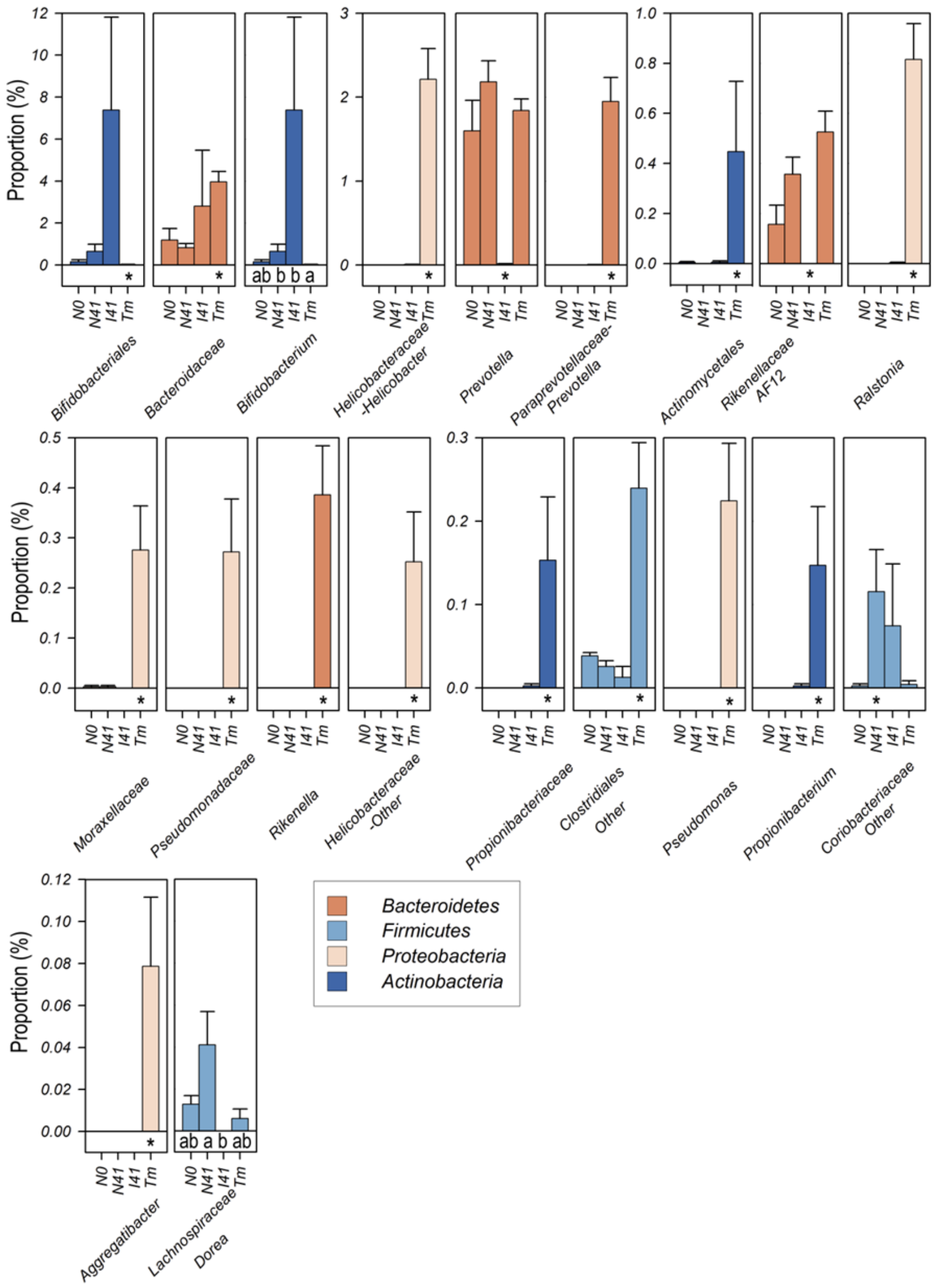

fig. S4. Community abundance differences were compared at all taxonomic levels to identify significant shifts between groups. Error bars are standard error of mean. * denotes samples are significantly different to all other samples, or shared letter indicate not significantly different. Results are from corrected post hoc Dunn Test after FDR correction on Kruskal-Wallis Test results (p values in database S2). All figures show means ± SEM.

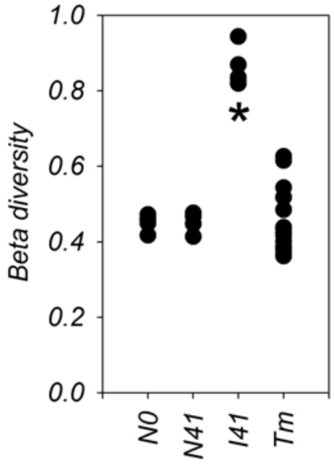

fig. S5. Increase in β diversity as a result of infection in the host cacal microbiota, not seen in *T. muris*. N0= Naïve cecal contents day 0, N41 = Naïve cecal contents day 41 p.i., I41 = Infected cecal contents day 41 p.i., Tm = *T. muris* microbiota day 41 p.i. β diversity is measured as an average Bray Curtis dissimilarity of each replicate compared to all other replicates within a group. * denotes samples are significantly different to all others tested by post hoc TukeyHSD on ANOVA results ($p < 0.001$). Samples were normally distributed.

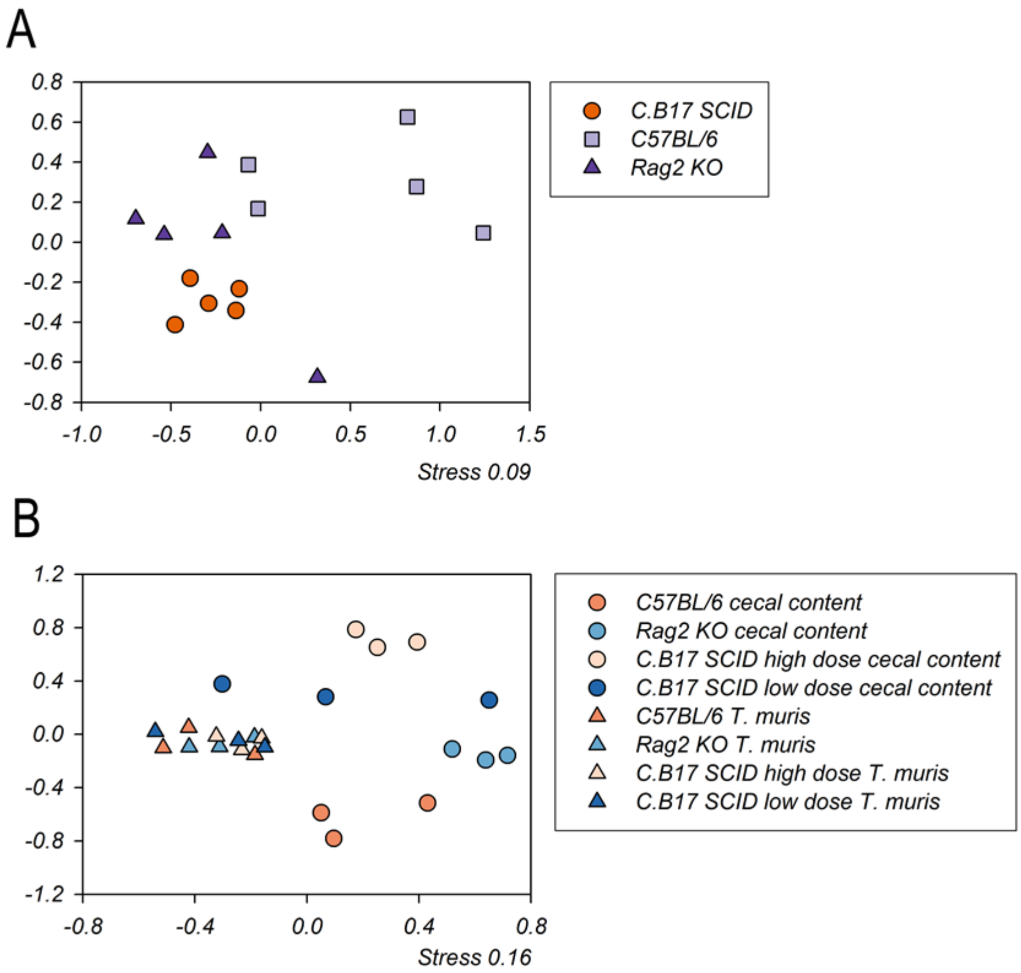

fig. S6. NMDS analysis of host intestinal microbiotas from different mouse strains infected with a high or low dose of *T. muris* compared to uninfected controls. NMDS analysis of the bacterial communities associated with (**A**) stool samples from naïve C.B17 SCID (n=5), C57BL/6 (n=5) and Rag2 KO (n=5) mice by 16S rRNA gene DGGE. All groups are significantly different to one another ($p < 0.02$), (**B**) bacterial communities from cecal contents and isolated *T. muris* adult worms from SCID, C57BL/6 and Rag2 KO mice (n=3 for all groups) by 16S rRNA gene DGGE. Each point represents an individual mouse or *T. muris* adult isolated from an individual mouse. *T. muris* samples are not significantly different from one another. Axis represents scale for Euclidian distance between samples centred on zero. Stress indicates the quality of fit of the data (< 0.2 is a good fit).

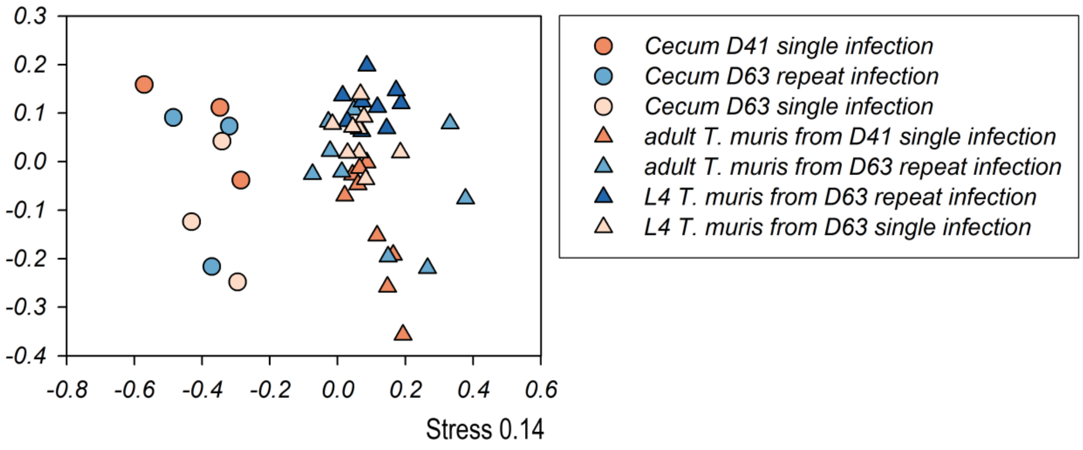

fig. S7. NMDS analysis of DGGE comparing the microbiota of *T. muris* isolated from C57BL/6 mice infected with a low dose of *T. muris* at day 0, day 41, or both days (a single and repeat infection). Each point represents an individual mouse or *T. muris* adult isolated from an individual mouse. All *T. muris* are not significantly different to one another. Axis represents scale for Euclidian distance between samples centred on zero. Stress indicates the quality of fit of the data (< 0.2 is a good fit).

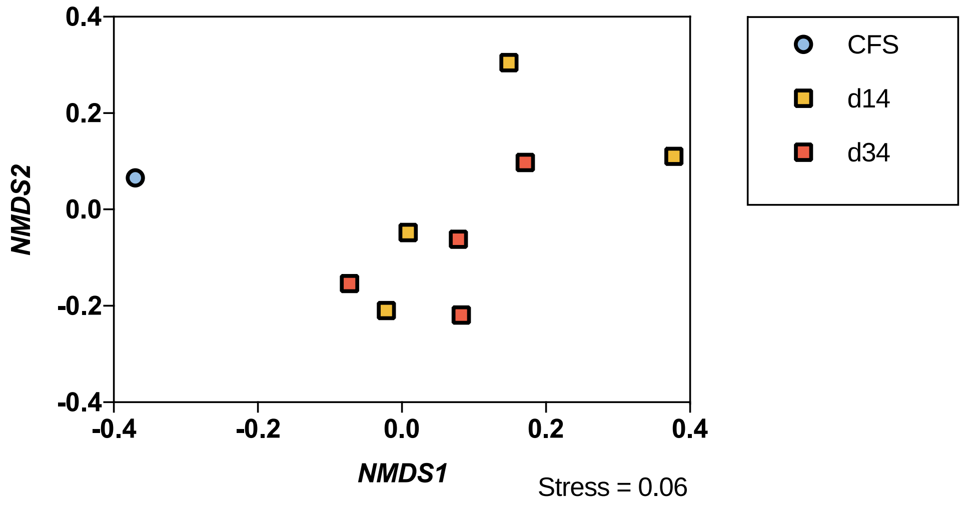

fig. S8. NMDS analysis of DGGE comparing the microbiota of GF mice that have been reconstituted with a cecal slurry from chronically infected C57BL/6 mice. Blue dot indicates

the inoculum (CFS), yellow squares are 4 individual mice at day 14 post-inoculation (d14) and red squares, the same mice at day 34 post-inoculation (d34). Axis represents scale for Euclidian distance between samples centred on zero.

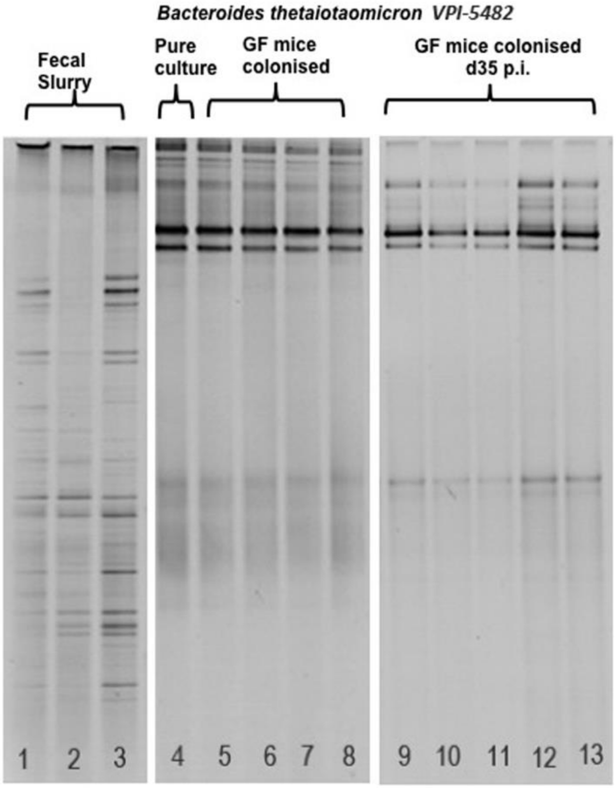

fig. S9. DGGE of fecal samples from GF mice inoculated with naïve mouse cecal slurry (lanes 1 to 3), pure culture of *Bt* strain VPI-5482 (lane 4), and GF mice inoculated with *Bt* (lanes 5 to 8) 12 days after inoculation and those inoculated with *Bt* at day 35 p.i. (lanes 9 to 13).

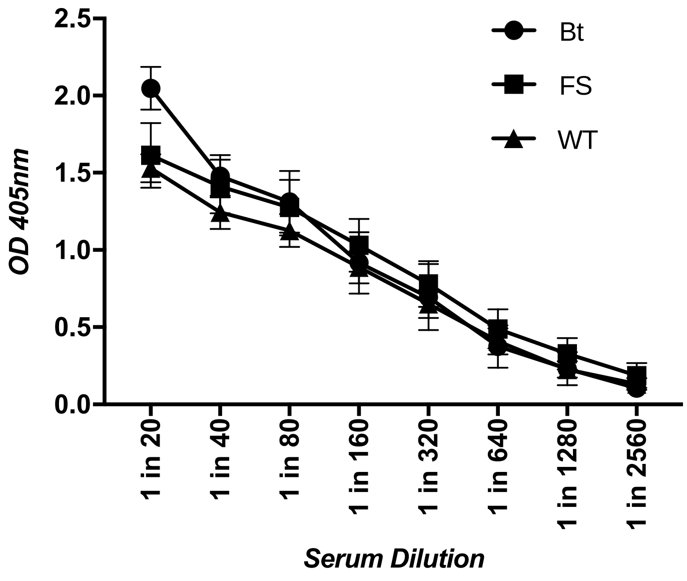

fig. S10. Parasite-specific IgG2a/c antibody in serum from low dose–infected GF mice that had been reconstituted with *Bt* strain VPI-5482, with a naïve FS from a WT C57BL/6 mouse and WT C57BL/6 control mice. Error bars are ± SEM.

table S1. Species shared between groups: naïve mice, infected mice, and *T. muris* microbiotas. Species identified in all samples in a group are marked with an X. Highlight indicates species are shared between groups.

table S2. *P* values and FDR-adjusted *P* values for differences in bacterial proportions at different taxonomic levels between groups. Posthoc Dunn tests were undertaken on significant results between naïve mice, infected mice and *T. muris* microbiotas.
